# Effect of postoperative radiotherapy for squamous cell cancer of the breast in a surveillance epidemiology and end results population-based study

**DOI:** 10.18632/oncotarget.7222

**Published:** 2016-02-06

**Authors:** San-Gang Wu, Jia-Yuan Sun, Wen-Ming Liu, Feng-Yan Li, Huan-Xin Lin, Zhen-Yu He

**Affiliations:** ^1^ Xiamen Cancer Center, Department of Radiation Oncology, The First Affiliated Hospital of Xiamen University, Xiamen, People's Republic of China; ^2^ Sun Yat-sen University Cancer Center, State Key Laboratory of Oncology in South China, Department of Radiation Oncology, Collaborative Innovation Center of Cancer Medicine, Guangzhou, People's Republic of China; ^3^ Department of Gastroenterology, Zhongshan Hospital of Xiamen University, Xiamen, People's Republic of China

**Keywords:** squamous cell cancer of the breast, breast cancer, radiotherapy, prognosis

## Abstract

The therapeutic value of postoperative radiotherapy (RT) for squamous cell cancer of the breast (SCCB) is unclear. This retrospective study used a population-based national registry to determine the impact of postoperative RT on survival of women with SCCB. The Surveillance Epidemiology and End Results (SEER) database was used to identify females with SCCB who underwent primary surgical resection from 1973 to 2012. Kaplan–Meier survival analysis and Cox regression proportional hazard methods were used to determine the impact of RT following resection associated with cause-specific survival (CSS) and overall survival (OS). A total of 523 patients met the eligibility criteria. The median follow-up time was 55 months, the 10-year CSS and OS rates were 65.6%, and 46.0%, respectively. A total of 167 patients (31.9%) received postoperative RT. Multivariate analysis indicated that advanced pT and pN stage, and no postoperative RT were independently associated with poor OS; advanced pT and pN stage were independently associated with poor CSS. Postoperative RT was significantly associated with improved 10-year OS (54.5% *vs.* 42.0%, *P* =.001), but had no effect on CSS (*P* =.217). Analysis of patients with different stages of SCCB indicated that RT was associated with improved CSS (*P* =.047) and OS (*P* <.001) in those with stage II cancer and improved OS in patients with stage pN0 cancer (*P* <.001). Postoperative RT improved the survival of SCCB patients, especially in those with stage II and stage pN0 cancer.

## INTRODUCTION

Squamous cell cancer of the breast (SCCB) is a rare type of breast cancer that accounts for approximately 0.04-0.1% of all breast cancers, and less than 0.1% of all invasive breast ductal carcinomas [1-4]. SCCB is diagnosed by exclusion of other more common cancers. In particular, the diagnosis requires that: *(i)* the tumor origin does not arise from the overlying skin, nipple, or adenexal components, *(ii)* more than 90% of the tumor consists of squamous cells, *(iii)* there is no evidence of ductal or mesenchymal elements within the tissue sample, and *(iv)* no other sites of primary squamous cell cancer are present [1, 2, 5-7]. Because of the rarity of this cancer, there is currently no consensus on the treatment and prognosis of these patients.

Many previous studies have shown that locoregional radiotherapy (RT) can improve cause-specific survival (CSS) and overall survival (OS) of female breast cancer patients [8-10], but there is limited research on the effect of RT in SCCB. Moreover, many of these previous studies were single-institution retrospective reviews with limited numbers of patients, so it is difficult to make recommendations for patients with different stages of SCCB. The ideal locoregional RT regimens for patients with different stages of SCCB are still uncertain. In this study, we analyzed the effect of postoperative RT on the survival of patients with SCCB using a population-based national registry, Surveillance, Epidemiology, and End Results (SEER).

## PATIENTS AND METHODS

### Patients

Data were obtained from the current SEER database, which consists of 18 population-based cancer registries of patients in the United States. SEER data are an open-access resource for cancer-based epidemiology and survival analyses. SEER*Stat software from the National Cancer Institute (Surveillance Research Program, National Cancer Institute SEER*Stat software, http://www.seer.cancer.gov/seerstat, version 8.2.1) was used to identify eligible patients. Patients with diagnoses of SCCB from 1973 to 2012 were identified. We obtained permission to access research data files with the reference number 11252-Nov2014 [11].

All included patients were females diagnosed with SCCB, received cancer-directed surgery, and had records on whether postoperative RT was used. Pathologic diagnosis was based on the primary site using the International Classification of Disease for Oncology, Third Edition (ICD-O-3). Use of the SEER database does not require informed consent. This study was approved by the ethics committee of the First Affiliated Hospital of Xiamen University (Xiamen) and Sun Yat-sen University Cancer Center (Guangdong).

### Clinicopathologic factors

The following clinical and pathologic factors were collected from the SEER database: age at diagnosis, race, grade, tumor stage, tumor size (pT), lymph node status (pN), estrogen receptor (ER) status, progesterone receptor (PR) status, human epidermal growth factor 2 (HER2) status, and use of adjuvant external beam RT. Survival, cause of death, and duration of follow-up were recorded.

### Statistical analysis

The χ^2^ and Fisher's exact probability tests were used to analyze differences in the qualitative data. Univariate and multivariate Cox regression analyses were used to identify factors that were significantly associated with CSS and OS. Multivariable analyses were performed for factors that were significantly associated with CSS and OS in the univariate analyses. Calculation of survival rates were plotted by the Kaplan-Meier method, and compared using the log-rank test. All data were analyzed using SPSS statistical software, version 21.0 (IBM Corporation, Armonk, NY, USA). A *P*-value less than .05 was considered statistically significant.

## RESULTS

### Patient characteristics and survival

A total of 523 patients met the eligibility criteria, 167 of whom (31.9%) received post-operative RT (Table 1). The median age was 66 years (range: 24-102 years). Among patients whose pT stage, pN stage, tumor stage, ER status, PR status, and HER2 status were known, 75.8% (294/388) had stage T2-T4 SCCB, and 73.6% (293/398) had negative lymph nodes. Stage I, II, III, and IV SCCB was present in 24.8% (102/412), 51.5% (212/412), 18.7% (77/412), and 5.0% (21/412) of patients, respectively. Of the 319, 315 and 50 patients whose ER, PR and HER2 status were available, respectively, a total of 81.2% (259/319) of patients were ER negative, 88.3% (278/315) were PR negative, and 92.0% (46/50) were HER2 negative. Patients who were older than 50 years (*P* = 0.002) and with more advanced cancer (*P* = .004) were more likely to have received postoperative RT (Table 1).

**Table 1:** Patient characteristics

Characteristic	n (%)	Without RT (%)	With RT (%)	*P*
Age (years)				
≤50	95 (18.2)	52 (14.6)	43 (25.7)	0.002
>50	428 (81.8)	304 (85.4)	124 (74.3)	
Race				
Black	64 (12.2)	43 (12.2)	21 (12.7)	0.943
White	435 (83.2)	297 (84.1)	138 (83.1)	
Other	20 (3.8)	13 (3.7)	7 (4.2)	
Unknown	4 (0.8)			
pT stage				
pT0-1	94 (18.0)	59 (23.9)	35 (24.8)	0.132
pT2	178 (34.0)	122 (49.4)	56 (39.7)	
pT3	72 (13.8)	44 (17.8)	28 (19.9)	
pT4	44 (8.4)	22 (8.9)	22 (15.6)	
Unknown	135 (25.8)			
pN stage				
pN0	293 (56.0)	196 (77.2)	97 (67.4)	0.152
pN1	74 (14.1)	39 (15.4)	35 (24.3)	
pN2	20 (3.8)	12 (4.7)	8 (5.6)	
pN3	11 (2.1)	7 (2.7)	4 (2.7)	
Unknown	125 (23.9)			
Metastasis				
M0	404 (77.2)	259 (93.5)	145 (98.0)	0.058
M1	21 (4.0)	18 (6.5)	3 (2.0)	
Unknown	98 (18.8)			
Stage				
I	102 (19.5)	69 (25.7)	33 (22.9)	0.004
II	212 (40.5)	143 (53.4)	69 (47.9)	
III	77 (14.7)	38 (14.2)	39 (27.1)	
IV	21 (4.0)	18 (6.7)	3 (2.1)	
Unknown	111 (21.3)			
Grade				
G1	50 (9.5)	36 (13.6)	14 (10.4)	0.212
G2	116 (22.2)	82 (31.1)	34 (25.2)	
G3-4	233 (44.6)	146 (55.3)	87 (64.4)	
Unknown	124 (23.7)			
ER status				
Negative	259 (49.5)	156 (79.2)	103 (84.4)	0.245
Positive	60 (11.5)	41 (20.8)	19 (15.6)	
Unknown	204 (39.0)			
PR status				
Negative	278 (53.2)	175 (89.7)	103 (85.8)	0.295
Positive	37 (7.1)	20 (10.3)	17 (14.2)	
Unknown	208 (39.7)			
HER2 status				
Negative	46 (8.8)	26 (89.7)	20 (95.2)	0.473
Positive	4 (0.8)	3 (10.3)	1 (4.8)	
Unknown	473 (90.4)			

G1, well; G2, moderately; G3, poorly; G4, undifferentiated; ER, estrogen receptor; PR, progesterone receptor; HER2, human epidermal growth factor 2.

**Figure 1:**
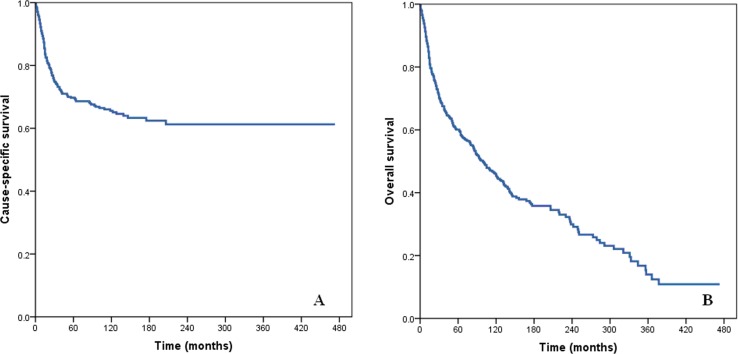
Cause-specific survival (A) and overall survival (B) of patients with squamous cell cancer of the breast

The median duration of follow-up was 55.0 months (range: 1-473 months). The 5-year and 10-year CSS rates were 69.7% and 65.6%, and the 5-year and 10-year OS rates were 60.1% and 46.0%, respectively (Figure 1A-1B).

### Analysis of prognosis

Univariate Cox survival analysis showed that patients who were black, had advanced pT stage, and advanced pN stage had significantly poorer CSS (Table 2). However, no significant differences in CSS were observed for patients with and without postoperative RT. Univariate analysis also indicated that patients who were older, black, had advanced pT stage, advanced pN stage, PR negative disease and did not receive postoperative RT had significantly poorer OS.

**Table 2:** Univariate analysis of cause-specific survival and overall survival

Characteristic	CSS			OS		
	HR	95%CI	*P*	HR	95%CI	*P*
Age (years)						
≤50	1			1
>50	1.453	0.941-2.244	0.092	2.953	2.009-4.234	<0.001
Race						
Black	1			1
White	0.557	0.369-0.839	0.005	0.641	0.463-0.888	0.007
Other	0.818	0.357-1.874	0.635	0.607	0.296-1.249	0.175
pT stage						
pT0-1	1			1
pT2	2.181	1.153-4.125	0.016	1.415	0.945-2.118	0.092
pT3	4.721	2.419-9.212	<0.001	2.801	1.789-4.385	<0.001
pT4	9.167	4.638-18.120	<0.001	4.405	2.682-7.208	<0.001
pN stage						
pN0	1			1
pN1	2.475	1.468-3.525	<0.001	1.732	1.209-2.483	0.003
pN2	4.090	2.292-7.298	<0.001	3.021	1.815-5.028	<0.001
pN3	4.657	2.131-10.177	<0.001	2.782	1.296-5.969	0.009
Grade						
G1	1			1
G2	1.228	0.621-2.430	0.555	0.803	0.526-1.227	0.311
G3-4	1.468	0.780-2.762	0.235	0.746	0.504-1.105	0.144
ER status						
Negative	1			1
Positive	1.228	0.749-2.015	0.415	0.984	0.645-1.504	0.942
PR status						
Negative	1			1
Positive	0.642	0.311-1.326	0.231	0.510	0.276-0.943	0.032
Radiotherapy						
No	1			1
Yes	0.807	0.573-1.137	0.220	0.650	0.497-0.849	0.002

CSS, cause-specific survival; OS, overall survival; HR, hazard ratio; CI, confidence interval; G1, well; G2, moderately; G3, poorly; G4, undifferentiated; ER, estrogen receptor; PR, progesterone receptor.

We used multivariate Cox analysis, with adjustment for significant factors from the univariate analysis, to assess the association of different parameters with CSS and OS (Table 3). The results show that advanced pT stage and advanced pN stage were independently associated with poorer CSS. Advanced pT stage, advanced pN stage, and no postoperative RT were independently associated with poorer OS.

**Table 3:** Multivariate analyses of cause-specific survival and overall survival

Characteristic	CSS			OS		
	HR	95%CI	*P*	HR	95%CI	*P*
Age (years)						
≤50	1			1		
>50	1.695	0.960-2.992	0.069	1.373	0.708-2.662	0.348
Race						
Black	1			1		
White	0.619	0.367-1.043	0.072	0.778	0.412-1.471	0.441
Other	0.638	0.212-1.921	0.424	1.783	0.561-5.665	0.327
pT stage						
pT0-1	1			1		
pT2	2.090	1.072-4.076	0.030	2.379	1.055-5.367	0.037
pT3	4.234	2.095-8.557	<0.001	4.804	2.022-11.412	<0.001
pT4	7.278	3.433-15.432	<0.001	9.494	3.775-23.878	<0.001
pN stage						
pN0	1			1		
pN1	1.553	0.953-2.529	0.077	2.064	1.178-3.619	0.011
pN2	3.406	1.851-6.267	<0.001	4.518	2.330-8.759	<0.001
pN3	2.652	1.188-5.919	0.017	3.064	1.256-7.464	0.014
PR status						
Negative	—			1		
Positive	—	—	—	0.482	0.215-1.083	0.077
Radiotherapy						
No	—			1		
Yes	—	—	—	0.439	0.266-0.723	0.001

CSS, cause-specific survival; OS, overall survival; HR, hazard ratio; CI, confidence interval; PR, progesterone receptor.

### The relationship of the postoperative RT and survival

**Figure 2:**
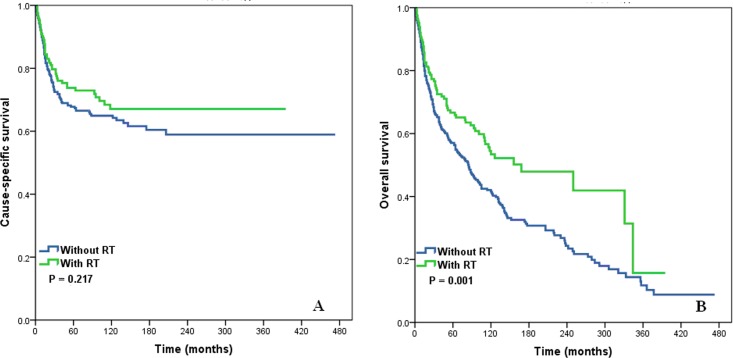
Cause-specific survival (A) and overall survival (B) of squamous cell cancer of the breast patients with and without post-operative radiotherapy

Kaplan-Meier analysis indicated that postoperative RT was significantly associated with better OS (log-rank test: *P* = .001) (Figure 2A). The 5- and 10-year OS rates were 66.7% and 54.5% for patients given RT, and were 57.0% and 42.0% for those not given RT. However, postoperative RT had no effect on CSS (log-rank test: *P* = .217) (Figure 2A-2B).

**Figure 3:**
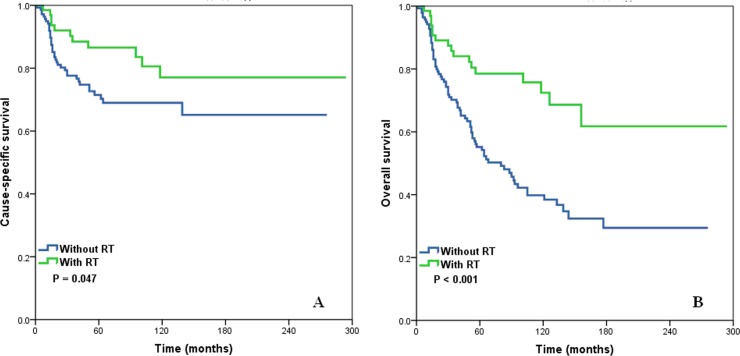
Cause-specific survival (A) and overall survival (B) of stage II squamous cell cancer of the breast patients with and without post-operative radiotherapy

We also determined the influence of postoperative RT on survival of patients with different stages of SCCB (Figure 3). The results indicate that RT was significantly associated with improved CSS (log-rank test: *P* = .047) and OS (log-rank test: *P* < .001) for patients with stage II SCCB (Figure 3A-3B). There were trends for improved CSS and OS for patients given RT who had stage I and stage III SCCB, but these were not statistically significant (log-rank test: *P* >.05) (Table 4).

**Table 4:** Cause-specific survival and overall survival by stage and radiotherapy

Stage	Median survival (months)	5-year		10-year		*P*
		Without RT	With RT	Without RT	With RT	
Tumor stage						
CSS						
I	—	85.9	100	83.8	95.5	0.062
II	—	71.5	86.6	69.0	77.1	0.047
III	34	33.8	46.7	0	35.6	0.327
IV	5	9.3	0	0	0	0.689
OS						
I	175	81.7	89.2	62.7	63.9	0.174
II	121	55.2	78.5	39.8	72.4	<0.001
III	28	30.6	46.7	18.3	35.6	0.327
IV	5	7.4	0	0	0	0.772
Nodal stage						
CSS						
pN0	—	75.0	84.0	73.1	76.0	0.163
pN1	—	51.8	61.1	39.9	61.1	0.248
pN2	34	23.4	50.0	—	—	0.463
pN3	22	28.6	33.0	—	—	0.486
OS						
pN0	123	61.4	78.2	44.9	65.9	<0.001
pN1	52	48.1	50.2	19.5	46.0	0.182
pN2	26	20.8	50.0	—	—	0.255
pN3	22	28.6	33.0	—	—	0.486

CSS, cause-specific survival; OS, overall survival; RT, radiotherapy.

**Figure 4:**
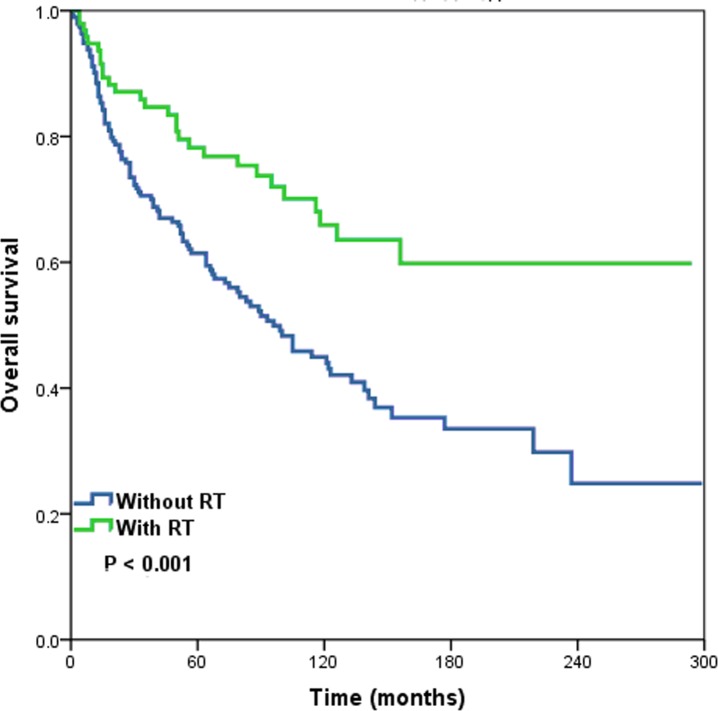
Overall survival of pN0 stage squamous cell cancer of the breast patients with and without post-operative radiotherapy

We also examined the prognostic effect of postoperative RT on the OS of patients with different pN stages of SCCB (Figure 4). The results indicate that RT was associated with significantly improved OS for patients with stage pN0 cancer (log-rank test: *P* < .001). RT also tended to increase the CSS in pN0-N3 stage patients and OS in pN1-N3 stage patients, although this was not statistically significant (log-rank test: *P* > .05) (Table 4).

## DISCUSSION

This is the largest retrospective analysis to assess the effect of postoperative RT on the survival of patients with SCCB. Our results indicated that RT improved the survival of SCCB patients, especially those with stage II cancer and no regional lymph node metastasis (pN0). Previous studies have shown that postoperative RT improves local control and survival in high-risk invasive breast cancers [8-10], but the present study is the first to identify a survival benefit for SCCB patients who receive postoperative RT.

Previous research indicated that SCCB is less likely to undergo lymphatic spread than adenocarcinomas. In particular, only 10 to 30% of SCCB patients have lymph node infiltration at the time of surgery [1, 12]. Case reports have indicated that SCCB is associated with high expression of Ki-67, a marker of proliferation [2, 13, 14]. Over 85% of patients are ER- and PR-, and most are also HER2- [4, 12, 15]. SCCB patients with the triple-negative subtype (ER-/PR-/HER2-) seem to have poor prognoses.[16] In this study, most patients were lymph node negative, ER-, PR-, and HER2-. The 10-year CSS and OS rates of our patients were 65.6% and 46.0%, respectively. Our results therefore underline the aggressive nature of SCCB, and suggest that comprehensive treatment should be considered for patients with potentially poor prognoses.

RT is provided as a standard of care for female breast cancer patients after breast conservation surgery, and is frequently given after mastectomy in high-risk patients [8-10, 17]. A previous SEER analysis showed that among 137 patients with SCCB from 1998 to 2001, only 35% received adjuvant RT, although the study did not analyze the effect of RT on survival [15]. Among 31 patients in the University of Texas M.D. Anderson Cancer Center with SCCB, 19 patients were treated with postoperative RT, and the recurrence-free survival (P = .210) and OS (*P* = .840) were not significantly different from those without RT.[15] Two Chinese studies examined 58 patients with SCCB, 12 of whom received postoperative RT, and reported that RT provided no significant survival benefit [18, 19]. Thus, SCCB seems to be relatively radioresistant, despite the fact that SCCs are generally considered to be radiosensitive [20]. In our cohort of 523 SCCB patients, only 167 patients (31.9%) received RT, even though 28.1% of the patients were lymph node-positive. Our results showed that postoperative RT was associated with improved OS, but had no effect on CSS. Thus, the present study is the first to identify a survival benefit for postoperative RT in patients with SCCB.

Our results found that RT specifically improved the survival of SCCB patients with stage II cancer and those with pN0 stage. The reasons for the beneficial effect of RT in these particular groups are not obvious. Four patients in the M.D. Anderson Cancer Center series who experienced a locoregional recurrence (LRR) had either T1N0 or T2N0 tumors [15]. Thus, the benefit of RT for patients with early-stage SCCB could in part be attributed to the aggressive nature of SCCB. Among our patients, the median survival time for those with stage III-IV and pN2-3 cancer was very short (less than 3 years). Thus, more obvious benefit of RT for stage II patients and pN0 stage with moderate risk of recurrence was observed. Our research and the previous study were both retrospective analyses [15], so the criteria used to select patients for postoperative RT remain unclear, but RT may not omit in management of locally advanced SCCB at presentation due to the absence of clinical trials.

Due to the limitations of the SEER database, we cannot identify risk factors for LRR of SCCB. Nayak et al. found that the 5-year LRR was 46% in 21 patients with SCCB, and the only statistically significant feature associated with LRR was the presence of a spindle cell component comprising >10% of the tumor (*P* = .006) [3]. The lack of response of SCCB to postoperative RT may reflect the mixed cell type present or the palliative use of RT to treat advanced disease.

SCCB is considered resistant to the standard chemotherapy regimens used for other breast cancers. Nevertheless, some studies found that adjuvant cisplatin-based regimens could be effective [6, 7, 21, 22]. In addition, 62.1% to 85.7% of SCCB patients have overexpression of epidermal growth factor receptor (EGFR) [15, 19, 23]. Although no study has shown that combined pharmacotherapy increases the sensitivity of SCCB to radiotherapy, studies of other cancers showed that a cisplatin-based regimen with an anti-EGFR agent may radiosensitize squamous cancer cells [24-27]. Future studies with large sample sizes are needed to investigate the effect of such regimens on the radiosensitivity of SCCB and the survival of SCCB patients.

The current study had several limitations that must be considered. The main limitations are the nonrandomized nature of the dataset and the inherent biases that exist in all retrospective studies. Second, the SEER database does not have data on the type and dose of systemic therapy, use of endocrine treatments, lymphovascular invasion, margin status, and local or regional recurrence. This hindered our ability to directly assess the effect of specific factors or clinical circumstances on outcome. In addition, this database provides little information of why clinicians administered or did not administer postoperative RT to different patients. Although retrospective reviews do not carry the power of prospective studies, no prospective studies have yet examined the effect of postoperative RT on SCCB.

In conclusion, postoperative RT was associated with improved survival of patients with SCCB, especially those with stage II cancer and pN0 stage patients. Prospective studies will be needed to confirm the results of this study and to establish the optimal protocols for use of RT in the management of SCCB.

## References

[1] Gupta C, Malani AK, Weigand RT, Rangineni G (2006). Pure primary squamous cell carcinoma of the breast: a rare presentation and clinicopathologic comparison withusual ductal carcinoma of the breast. Pathol Res Pract.

[2] Comellas N, Marin Gutzke M (2009). Primary pure squamous cell carcinoma of the breast presenting as a breast abscess. J Plast Reconstr Aesthet Surg.

[3] Nayak A, Wu Y, Gilcrease MZ (2013). Primary squamous cell carcinoma of the breast: predictors of locoregional recurrence and overall survival. Am J Surg Pathol.

[4] Grenier J, Soria JC, Mathieu MC, Andre F, Abdelmoula S, Velasco V, Morat L, Besse B, Dunant A, Spielmann M, Delaloge S (2007). Differential immunohistochemical and biological profile of squamous cell carcinoma of the breast. Anticancer Res.

[5] Lee ES, Lundberg TM, Rose JF, Ley MB, Waer A, Livingston RB, Stopeck AT, Chalasani P, Gonzalez VJ, LeBeau LG, Viscus RK (2014). A Retrospective Case Series and Literature Review of Primary Squamous Cell Cancer of the Breast. J Surgery.

[6] Dejager D, Redlich PN, Dayer AM, Davis HL, Komorowski RA (1995). Primary squamous cell carcinoma of the breast: sensitivity to cisplatinum-based chemotherapy. J Surg Oncol.

[7] Stevenson JT, Graham DJ, Khiyami A, Mansour EG (1996). Squamous cell carcinoma of the breast: a clinical approach. Ann Surg Oncol.

[8] Overgaard M, Hansen PS, Overgaard J, Rose C, Andersson M, Bach F, Kjaer M, Gadeberg CC, Mouridsen HT, Jensen MB, Zedeler K (1997). Postoperative radiotherapy in high-risk premenopausal women with breast cancer who receive adjuvant chemotherapy. Danish Breast Cancer Cooperative Group 82b Trial. N Engl J Med.

[9] Overgaard M, Jensen MB, Overgaard J, Hansen PS, Rose C, Andersson M, Kamby C, Kjaer M, Gadeberg CC, Rasmussen BB, Blichert-Toft M, Mouridsen HT (1999). Postoperative radiotherapy in high-risk postmenopausal breast-cancer patients given adjuvant tamoxifen: DanishBreast Cancer Cooperative Group DBCG 82c randomised trial. Lancet.

[10] Ragaz J, Olivotto IA, Spinelli JJ, Phillips N, Jackson SM, Wilson KS, Knowling MA, Coppin CM, Weir L, Gelmon K, Le N, Durand R, Coldman AJ, Manji M (2005). Locoregional radiation therapy in patients with high-risk breast cancer receiving adjuvant chemotherapy: 20-yearresults of the British Columbia randomized trial. J Natl Cancer Inst.

[11] Surveillance, Epidemiology, and End Results (SEER) Program (www.seer.cancer.gov) SEER*Stat Database: Incidence - SEER 18 Regs Research Data + Hurricane Katrina Impacted Louisiana Cases, Nov 2014 Sub (1973-2012 varying) - Linked To County Attributes - Total U.S., 1969-2013 Counties, National Cancer Institute, DCCPS, Surveillance Research Program, Surveillance Systems Branch, released April 2015, based on the November 2014 submission.

[12] Behranwala KA, Nasiri N, Abdullah N, Trott PA, Gui GP (2003). Squamous cell carcinoma of the breast: clinico-pathologic implications and outcome. Eur J Surg Oncol.

[13] Murialdo R, Boy D, Musizzano Y, Tixi L, Murelli F, Ballestrero A (2009). Squamous cell carcinoma of the breast: a case report. Cases J.

[14] Shui R, Li A, Yang F, Zhou X, Yu B, Xu X, Yang W (2014). Primary squamous cell carcinoma of the breast with unusual basal-HER2 phenotype. Int J Clin Exp Pathol.

[15] Hennessy BT, Krishnamurthy S, Giordano S, Buchholz TA, Kau SW, Duan Z, Valero V, Hortobagyi GN (2005). Squamous cell carcinoma of the breast. J Clin Oncol.

[16] Honda M, Saji S, Horiguchi S, Suzuki E, Aruga T, Horiguchi K, Kitagawa D, Sekine S, Funata N, Toi M, Kuroi K (2011). Clinicopathological analysis of ten patients with metaplastic squamous cell carcinoma of the breast. Surg Today.

[17] Clarke M, Collins R, Darby S, Davies C, Elphinstone P, Evans V, Godwin J, Gray R, Hicks C, James S, MacKinnon E, McGale P, McHugh T, Peto R, Taylor C, Wang Y (2005). Early Breast Cancer Trialists' Collaborative Group (EBCTCG). Effects of radiotherapy and of differences in the extent of surgery for early breast cancer on local recurrence and 15-year survival: an overview of the randomised trials. Lancet.

[18] Liu J, Yu Y, Sun JY, He SS, Wang X, Yin J, Cao XC (2015). Clinicopathologic characteristics and prognosis of primary squamous cell carcinoma of the breast. Breast Cancer Res Treat.

[19] Wang J, Zhang X, He J, Yang M, Tang J, Li X, Tang H, Xie X (2014). Co-expression of EGFR and CK5/6 in primary squamous cell carcinoma of the breast. Med Oncol.

[20] Wargotz ES, Norris HJ (1990). Metaplastic carcinomas of the breast. IV. Squamous cell carcinoma of ductal origin. Cancer.

[21] Gupta N, Vashisht R, Nimbran V, Gupta R, Dhingra N, Bhutani A (2012). Primary squamous cell carcinoma of the breast: case report and management decisions. J Cancer Res Ther.

[22] Sheela CS, Ramakant P, Shah G, Chandramohan V, Abraham D, Paul MJ (2013). Primary squamous cell carcinoma of breast presenting as a cystic mass. J Postgrad Med.

[23] Reis-Filho JS, Milanezi F, Carvalho S, Simpson PT, Steele D, Savage K, Lambros MB, Pereira EM, Nesland JM, Lakhani SR, Schmitt FC (2005). Metaplastic breast carcinomas exhibit EGFR, but not HER2, gene amplification and overexpression:immunohistochemical and chromogenic in situ hybridization analysis. Breast Cancer Res.

[24] Sano D, Matsumoto F, Valdecanas DR, Zhao M, Molkentine DP, Takahashi Y, Hanna EY, Papadimitrakopoulou V, Heymach J, Milas L, Myers JN (2011). Vandetanib restores head and neck squamous cell carcinoma cells' sensitivity to cisplatin and radiation *in vivo* and *in vitro*. Clin Cancer Res.

[25] Tanaka T, Yukawa K, Umesaki N (2005). Radiation enhances cisplatin-sensitivity in human cervical squamous cancer cells *in vitro*. Eur J Gynaecol Oncol.

[26] Zhao L, He LR, Xi M, Cai MY, Shen JX, Li QQ, Liao YJ, Qian D, Feng ZZ, Zeng YX, Xie D, Liu MZ (2012). Nimotuzumab promotes radiosensitivity of EGFR-overexpression esophageal squamous cell carcinoma cells byupregulating IGFBP-3. J Transl Med.

